# Striving for a silent knee: a qualitative study of patients’ experiences with knee replacement surgery and their perceptions of fulfilled expectations

**DOI:** 10.1080/17482631.2019.1620551

**Published:** 2019-05-22

**Authors:** Josefina Skogö Nyvang, Margareta Hedström, Maura D. Iversen, Sissel Andreassen Gleissman

**Affiliations:** a Department of Clinical Science, Intervention and Technology, Division of Orthopaedics and Biotechnology, Karolinska Institutet, Huddinge, Sweden; b Department of Orthopaedics, Karolinska University Hospital Huddinge, Stockholm, Sweden; c Department of Women and Children’s Health, Karolinska Institutet, Elevhemmet H2:00, Karolinska University Hospital Solna, Stockholm, Sweden; d Department of Physical Therapy, Movement & Rehabilitation Sciences, Northeastern University, Brigham & Women’s Hospital, Division of Rheumatology, Immunology & Allergy, Section of Clinical Sciences, and Department of Medicine, Harvard Medical School, Boston, MA, USA; e Department of Nursing Science, Sophiahemmet University, Stockholm, Sweden

**Keywords:** Knee arthroplasty, knee osteoarthritis, qualitative research, experiences, outcome, satisfaction

## Abstract

**Purpose:** Fifteen to twenty percent of patients with a knee arthroplasty are dissatisfied with their replaced joint. This study aimed to describe patients’ experiences of undergoing knee replacement surgery, both total- and unicompartmental knee replacement, and post-operative recovery, and to determine whether expectations of surgery were fulfilled.

**Methods:** Using semi-structured interviews, this study describes twelve patients’ experiences of undergoing knee replacement surgery in the prior year, their post-operative recovery, and whether their expectations of surgery were fulfilled. Qualitative thematic analysis was used.

**Results:** A theme “striving for a silent knee”, and two categories “the bumpy road to recovery” and “the presence of the future” were created. Some participants were not fully restored one year after surgery. Those still in pain had thoughts about the future, from hoping to improve, to accepting living with an aching knee. Those with no pain, did not think about their knee—the knee had become silent.

**Conclusions:** Surgeons often inform patients that the recovery time after a knee arthroplasty is one year, which in light of this study, might be too short. We suggest that a follow-up after one year might identify those who need enhanced physical and psychological support to get the best possible outcome, whether it is to help patients accepting persistent symptoms or to continue striving towards a silent knee.

## Introduction

Knee-related pain and functional disabilities are the main indications for performing knee arthroplasty among patients with osteoarthritis (OA). However, 15–20% of the patients receiving a knee arthroplasty report being dissatisfied with their replaced joint and experience persistent pain and limited function (Wylde, Dieppe, Hewlett, & Learmonth, 2007). The discrepancy between patient-perceived surgical outcomes and the surgeon’s perception of a good outcome has been described and can be associated with differences in pre-operative expectations between patients and surgeons (Ghomrawi et al., 2011; Noble, Fuller-Lafreniere, Meftah, & Dwyer, 2013). The use of patient-reported outcomes (PROs) in patient-centred care helps providers to understand the patients’ symptoms before and after surgery. The Swedish Knee Arthroplasty Registry (SKAR) recently incorporated PROs in the registry (Swedish Knee Arthroplasty Registry [SKAR], 2016). However, information obtained from PROs and gathered during patients’ interviews are not always concordant (Brédart, Marrel, Abetz-Webb, Lasch, & Acquadro, 2014; Woolhead, Donovan, & Dieppe, 2005). The ultimate goal when performing a knee replacement is improved function, reduced pain and ideally patients’ 100% satisfaction with the surgical outcome. Understanding patients’ experiences of undergoing surgery and associated post-operative recovery may improve management and ultimately lead to better long-term outcomes. As a complement to pre-operative examination and data derived from PROs, acknowledging the patient’s individual experiences of living with knee OA and their expectations for life after surgery may enhance patient care (Nyvang, Hedström, & Andreassen Gleissman, 2016). In-depth interviews can provide a foundation for future quantitative and qualitative research and offer new perspectives to facilitate a greater understanding of how patients perceive a good or poor post-surgical outcome. Additionally, satisfaction with care and perception of fulfilled expectations are multifaceted outcomes with no good objective tools to measure why qualitative studies are needed.

### Aim

This study aimed to describe patients’ experiences of undergoing knee replacement surgery, both total- and unicompartmental knee replacement, and post-operative recovery, and to determine whether expectations of surgery were fulfilled.

## Patients and methods

### Study design

This study used a qualitative descriptive design with an inductive approach (Patton, 2015) to comprehensively describe patients’ perspectives and to gather rich information about their experiences of undergoing knee replacement surgery and post-operative rehabilitation and identify the extent to which their expectations of surgery were met.

### Research team

We gathered a research team who had different pre-understanding and experiences of joint replacement based on their varied clinical backgrounds in orthopaedics (JSN, MH), nursing (SAG) and physiotherapy (MI) as well as their research on outcomes following joint arthroplasty and long-term pain.

### Patients

**Figure 1.**
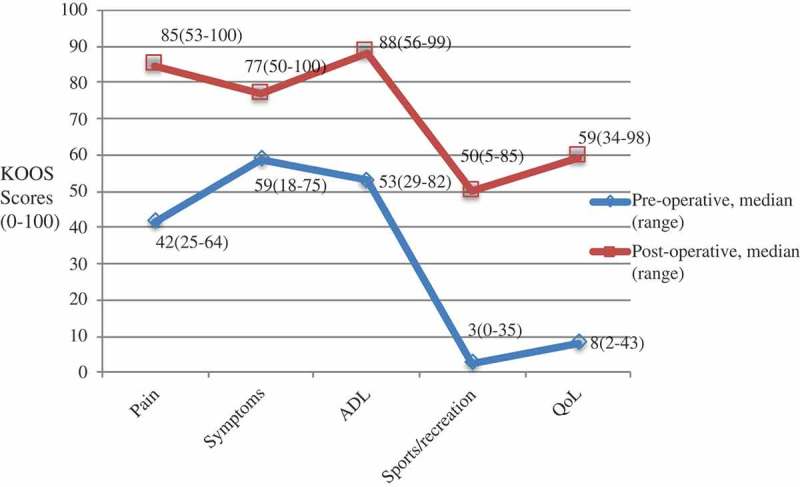
KOOS subscales before a knee arthroplasty and at one-year follow-up for the twelve patients included in the study. *KOOS: Knee Injury and Osteoarthritis Outcome Score. ADL: Activities in Daily Living. QoL: Quality of Life*. The questionnaire is divided into five subscales: Symptom; Pain; Function in daily living (ADL); Function in sport and recreation; Knee-related Quality of Life (Knee-related QoL). Each subscale generates a final score ranging from 0–100 where 0 represent “worst” and 100 “best”.

Participants enrolled in this study met the following inclusion criteria: scheduled for knee replacement surgery at Karolinska University Hospital, Stockholm, Sweden, secondary to OA and could speak Swedish well enough to be interviewed in Swedish. Purposeful sampling was used to obtain variation in sex and age. Twelve patients who met the inclusion criteria were asked to participate in two interviews: the first occurred within one month prior to scheduled knee replacement surgery, previously published (Nyvang et al., 2016), and the second interview occurred one year after surgery. This paper describes data from the follow-up interview. One year post-operatively, contact was re-established over telephone by JSN with the participants and again asked if they were willing to perform the second interview and all agreed. Of the 12 patients, 2 received bilateral knee replacements (Table I). All patients completed the Knee Injury and Osteoarthritis Outcome Score (KOOS) questionnaire (Roos, Roos, Ekdahl, & Lohmander, 1998), a reliable and valid knee-specific PRO (Figure 1) (Steinhoff & Bugbee, 2014), a visual analogue scale (VAS) regarding self-assessed overall health (EQ-5D VAS) (Brooks, Jendteg, Lindgren, Persson, & Bjork, 1991) and a VAS for pain (Table I) (Price, McGrath, Rafii, & Buckingham, 1983), both prior to and one year after surgery. These measurements are routinely used in clinical practice by orthopaedic surgeons in the assessment of knee symptoms, general health, and pain. Longitudinal differences were not calculated since these scores solely were obtained to describe the study group.

**Table I.** Patient demographics for the twelve patients who had undergone a knee arthroplasty (n = 12).

Age, median (range)	67.0 (48–78)
Women	7
Total knee arthroplasty*	10
Unicompartmental arthroplasty*	2
Occupancy	
Retired	9
Working	3
EQ-5D VAS, median (range)	
Pre-operative	75 (30–93)
Post-operative	78 (34–98)
Pain VAS, median (range)	
Pre-operative	44 (4–76)
Post-operative	0 (0–43)

*One patient received bilateral total knee arthroplasties and one received bilateral unicompartmental knee arthroplasties during the year but not simultaneous. EQ-5D: EuroQol 5 dimensions. VAS: Visual Analogue Scale. EQ-5D VAS ranges from 0 to 100 where 100 is best possible health. Pain VAS ranges from 0 to 100 where 0 is no pain and 100 is worst thinkable pain.

### Data collection and procedure

This study used semi-structured qualitative interviews and the research team collaboratively developed an interview guide based on their clinical expertise, the literature on knee arthroplasty and post-operative recovery as well as from data gained during the pre-operative interviews (Nyvang et al., 2016). The interview guide included topics on: the participants’ experiences of the surgical procedure and related events, their experiences regarding post-surgical treatment and pain, post-operative recovery and physiotherapy, their perceived physical function and social relationships one-year post-surgery in relation to their pre-operative status, and their perception of fulfilled surgical expectations. Interviews started with the question: “Can you tell me about your experiences regarding the knee replacement surgery and the period thereafter?” All interviews were conducted in Swedish by JSN, who also interviewed the participants pre-operatively (Nyvang et al., 2016), which allowed the participants to feel comfortable as mutual trust was already established, leading to a relaxed interview. The interviewer (JSN) is a medical doctor, which was known by the participants, but had no involvement in the surgery or clinical follow-up and had not encountered the participants in her medical role. An interview guide was used as a support to ensure that all topics were covered but the participants were able to expand on a topic or introduce a new topic if it was meaningful to their stories and corresponded to the aim. The interviewer used in-depth probing, but not leading, questions to ensure the information was exhaustive and understood correctly. Between October 2014 and May 2015, the interviews took place at a location participants’ felt was convenient: two in the participants’ home, one in a separate place at a theatre foyer and nine at the hospital. All interviews were recorded and lasted between 26 and 63 min (mean = 43 min). In total, twelve participants were included in this study and their stories provided rich information and thus fulfilled this study’s needs.

### Data analysis

Qualitative thematic analysis was used to analyze the findings, which is considered a suitable approach for research questions in health and well-being (Braun & Clarke, 2014) and for describing a phenomenon experienced by persons living with a long-term illness (Braun & Clarke, 2006). All interviews were transcribed *verbatim* by a research assistant and then read through by JSN, while listening to the recording, to ensure good quality. In the first, descriptive part of the analysis, transcripts were read through several times by all authors, with the aim to comprehend the overall meaning of the interviews. Notes were made and important key phrases were highlighted and grouped into preliminary categories by JSN. The categories were then compared and discussed back and forth between all authors to reach consensus and to ensure internal validity (Patton, 2015). As a support for visualizing patterns and to highlight connections between key phrases and categories JSN used “messy maps” (Clarke, 2005) and MAXQDA Standard 12 software to highlight relevant text and arrange categories. “Messy maps” were used early in the analysis where relevant text was randomly scattered on a white page. Thereafter, lines were drawn to illustrate connections between different phrases. In this way, key phrases that got many lines were considered important and required further investigation (Clarke, 2005). During the interpretive analysis, transcripts were read again with the aim and categories in mind, and with a more interpretive mind-set to create a theme. Alongside reading and creating categories, writing the results was used as a part of the interpretive analysis in order to structure the findings and to visualize data (Bazeley, 2009). Thematic analysis requires an integrative process between the analytical steps and frequent discussions among the research team members to ensure trustworthiness (Braun & Clarke, 2006).

### Ethical considerations

All patients were informed of the voluntary participation and were ensured confidentiality. They gave their written informed consent to participate in two interviews, in accordance with the Declaration of Helsinki (World Medical Association, 2013). However, the participants were contacted by JSN and again asked if they were willing to participate in the second interview. The right to withdraw their consent was stressed and none declined. The Regional Ethical Review Board in Stockholm approved the study. Dnr: 2012/786–31/2.

## Results

The overriding theme, categories and subcategories as well as examples of the analytic process are presented in Table II.

**Table II.** Examples of the analytic process.

*Key phrases*	Code	Subcategory	Category	Theme
*I felt powerless. I had. I couldn’t influence [the care], others did the assessment. But I was in pain.*	Not satisfied with given care	Participation in care	***The bumpy road to recovery***	**Striving for a silent knee**
*I wished for more information, maybe from the physiotherapist or from the surgeon, of what to expect.*	Lack of information	Mental preparation		
*I don’t even think about the fact that I have had a knee replacement surgery*	Unawareness of the knee		***The presence of the future***	
*I have accepted my situation. I train and try to make it [the knee] a little better for each day. That’s all I can do.*	Acceptance			

## Striving for a silent knee

The journey from surgery towards recovery was reported to be hard and full of obstacles. These obstacles consisted of surgical complications, medication side effects or uncertainty that the knee was recovering properly. Patients remarked there were different facilitators to help overcome these obstacles. These facilitators included caregivers or relatives, or from the participant’s his or herself e.g., being positive or having persistence. Sometimes caregivers could become an obstacle, rather than a facilitator, if the participants felt their symptoms were not being taken seriously. Some facilitators helped the participants mentally prepare for what to expect during the recovery period, helping them see the anticipated future more clearly. Those whose pain persisted one year after surgery had many thoughts about how to make the future better. Some were still hoping to become better, with less pain and improved function, while others had started to accept living with an aching knee. Among those who no longer had pain, the future they expected pre-operatively was now present, letting them continue the life they had lived prior to OA—the knee had become silent.

### The bumpy road to recovery

The path to recovery contained obstacles and facilitators that helped the participants overcome difficulties. The participants felt well prepared of what to expect during the recovery period if they were able to participate in the given care, as desired. However, the participants could also meet resistance, which made them feel left out of care, and haltered their mental preparation. “The bumpy road to recovery” was divided into two subcategories: “participation in care” and “mental preparation”.

#### Participation in care

Participants did not always expect to participate in their medical care. Some said they trusted the caregivers’ judgment and professionalism and therefore, did as they were told rather than provide suggestions. Those participants were generally satisfied with their participation in their medical care. Some comments reflecting this attitude were:

‘No, but I listen carefully to what the doctor, I mean the experts, have to say. So, that is what you should do. I can’t sit there and object to what they suggest. So, no, but it’s … I was participating. I got to participate in rehabilitation and that.’

’During the surgery I was almost asleep, so I couldn’t participate [in the care]. And the aftercare, well, it was, when I came up to the ward, or down from surgery, where I spent one, or was it two days I lay there? It wasn’t much to be participating in.’

Some participants had higher demands for participation in their care, especially if complications or problems occurred. Then they wished to be included in how the problems should be solved; otherwise they felt left out and would not be satisfied with the care given.

’I felt, I felt powerless. I had. I couldn’t influence [the care], others did the assessment. But I was in pain.’

Further, when participants experienced a problem with their knee, some expressed a feeling of not being taken seriously or feeling left out when attempting to participate in their care. Sometimes this interaction resulted in a lack of problem resolution. For example, some experienced severe side effects from painkillers, such as vomiting, nausea or constipation, but were not offered an alternative. Those participants discontinued taking pain medication and did not get good pain relief.

’And I didn’t tolerate the morphine after the surgery. I vomited. And they [the caregivers] just answered: “you just have to vomit and take those painkillers”.’

Post-operative physiotherapy was offered to the participants with a goal to stand on their feet the same day as the surgery or the day after, at the latest. Participants were told to walk as much as possible, and the outcomes of this behaviour varied among participants. Some participants overstrained their knee, leading to exacerbation of their pain and knee swelling.

‘There [at the ward], everybody—from doctors, to nurses, to physiotherapists and everyone said that: “At this point you should move as much as you possibly can”. So that is what I did. But after two days, I think it was, the leg started to swell.’

Some participants expressed a desire to have their physiotherapy individualized to prevent overstraining incidents or to reduce the risk of performing movements that would hurt the knee. The amount of physiotherapy received after discharge from the hospital varied. Some participants felt the training they received from the physiotherapist was appropriate and good while others felt they were better off training by themselves. Some wished to participate in group physiotherapy with other patients who had knee replacements to help motivate them. The participants believed that post-operative training of the knee was necessary to get the best possible outcome after surgery.

#### Mental preparation

Some participants received information about the procedure from different sources such as relatives, health care providers, and their own previous joint arthroplasty experiences. These individuals felt well prepared for surgery and the post-operative period. As a result, they did not feel that their experiences (e.g., such as pain or difficulties walking) during the recovery period came as a surprise.

’I am very pleased with everybody and I understood exactly how everything was going to be. So, I was well prepared long before [the surgery], so to say. There were no surprises at all.’

Those participants who had a relative or friend who had undergone the same procedure turned to those individuals to verify that the rehabilitation was going in the right direction. This was more common than talking to the physiotherapist or the surgeon. Some participants stated they had more confidence in someone who had gone through the procedure him or herself.

’No, but I knew, my brother had, he told me how it was, how much pain it would be and I had spent time, I spent time with him when he got back from the surgery. Well, he told me, so I was well prepared. Well, he is quite like myself when it comes to medication and the like. Yes. No, so I had, I understood exactly how it was going to be and that was how it became.’

Some participants expressed a lack of information about the post-operative recovery making them feel uncertain about the healing process, what symptoms to expect and how much pain was normal after knee replacement surgery. If the participants experienced inexplicable symptoms or pain that their sources could not explain, they worried that they had done something wrong that jeopardized the positive outcome of the surgery. Some wished for more guidance of what to expect during their post-operative period—what was normal to feel and what was not.

’But when I didn’t really understand [if it was going the right way] you searched the Internet. How fast should it really go? How does other people experience this? So, there was a time when I doubted that it was going the right way. But it was.’

’I haven’t met very many who have undergone knee surgery so I don’t know. I don’t have anyone to compare to.’

The participants used strategies to overcome obstacles. Some considered themselves positive thinkers or having a persistent personality that helped them remain optimistic about the outcome.

‘It [the pain] got worse. It was a bit too much. But, well. I thought: “I have to take this”. Because that feeling, it overpowered the other [pain].’

Other strategies were related more to practicalities such as getting the knee checked out if it did not feel right.

### The presence of the future

Some participants were informed that their knee would be fully restored one year after the surgery. For some participants, the anticipated future did arrive one year after surgery, meaning that they had become pain free and were able to take long walks again. Hence, when satisfied and without pain the participants did not mention the future much. For others who had persistent pain and limited function, the future they had anticipated was yet not present and they tried to manage their expectations in order to accept that they might not be pain-free. For those who were not fully satisfied with their surgery, their thoughts ranged from still hoping that the knee would become better to accepting that the knee probably would not be as good as they had hoped.

‘But I had hoped a lot about this operation because, that I would be able to do a lot more things than I can do now. But I might get there. I hope so.’

‘I don’t want to have pain and I want to be able to lie on my knees. I want to be able to do that. Everything. Everything that you ever would want to do. That is what I would like to do, but I will probably never be able to do that. You have to realize that this is, like, a handicap that you just have to live with.’

Some participants were eager to engage in different tasks despite their pain and limited function and refused to be negatively affected by their knee.

‘My fear is to be sitting behind four walls. So that helped me to get out quickly. I was, I had to. I can’t just sit inside, I mean, talk to the cat and the walls. That doesn’t give me anything.’

One participant was not fully satisfied and was still hoping to get better. However, this person had a mind-set that was striving towards acceptance by learning to live with the new knee.

‘I have learnt to live with it but I haven’t really accepted it. I think you can put it like that. Because I, I dream that it will be as good as the left [knee]’.

The participants that were satisfied with their replaced knee did not talk much about the future in their interviews—the future was already present. Those who had few remaining symptoms were not always aware of their knee and could forget that they had an artificial knee joint. They had reached the point where the knee had become silent, with little pain and no longer considered the knee as an obstacle in their lives.

‘I don’t even think about the fact that I have had a knee replacement surgery.’

’Well, it [life] has changed in the way that I no longer hesitate to walk and like that, as I did before. You feel, you feel more alert and younger when you don’t have pain. And like. Limping is no fun, it feels good to not having to do that anymore.’

Participants were not always aware of their new joint, it emerged when they could not perform different movements, when the knee hurt, or when they heard clunking sounds coming from the knee. Those who had recurring symptoms were more aware of their knee and saw the knee as an alien body part.

‘I can feel the knee all the time, you know. Still it feels like an alien body part.’

Some participants could not say if their expectations had been met but were generally satisfied with their new joint.

‘But now since, since the ache is gone now and that is wonderful. I can play two rounds of golf and then sleep throughout the night [without pain] anyways. Unbelievable.’

However, some participants felt they were not fully restored one year after surgery, despite being satisfied or not. There was always more rehabilitation to be done to maximize the outcome.

’And now I get, I get almost surprised every day, even now, because I, I get further and further at the gym and you can do bigger movements and no, no, it’s going, it’s getting better and better all the time. And that’s what is so strange since it is over a year ago now but still it is getting better’.

Some participants said that their typical OA pain was gone but it had been replaced by a new type of pain. However, those who still had pain or limited function thought it was worth doing the surgery as they had done whatever they could to relieve their symptoms.

‘Yes, of course I thought that it [the knee] would be much better. But of course, it was worth the trouble to do the operation, it was. Absolutely! Otherwise I would have walked around and have been in even more pain maybe. With even more immobility than I have now. Because osteoarthritis is not a fun thing to have either, right?’

## Discussion

This study aimed to describe the experiences of patients undergoing knee replacement surgery and its associated post-operative rehabilitation, and to determine whether their expectations of surgery were met. The main finding was that if the participants experienced pain relief and improved knee-related function post-operatively, the knee had become silent and they could continue living their life as they had lived it before the onset of OA. For those who had persistent pain and loss of function, their thoughts ranged from holding onto hope that the knee would improve to accepting they might have to live a life with an always-noticeable knee. The overriding theme “Striving for a silent knee” represents the ultimate goal for all participants.

As many as 15–20% of individuals receiving a total knee arthroplasty report dissatisfaction with their replaced knee joint (Wylde et al., 2007). One previous study concluded that patients’ perceptions of surgical outcome corresponded to their pre-operative expectations of surgery and that there is a discrepancy between the level of expectations between surgeons and patients, where patients expect a better functional outcome (Noble et al., 2013). From the pre-operative interviews, we concluded that participants reported varying expectations of surgery but consistently expected to be able to walk without pain (Nyvang et al., 2016). Better communication between patients and providers and an individual assessment of the patients’ expectations has been suggested to improve satisfaction with care (Noble et al., 2013; Nyvang et al., 2016).

Patients with a knee arthroplasty, who suffer from persistent pain 2–5 years post-operatively, express emotions ranging from struggling to cope with pain towards accepting a life in pain (Jeffery, Wylde, Blom, & Horwood, 2011). The participants in this study had not yet reached the phase of acceptance; they were still hoping to see improvements in their pain and function. The standard assessment of final surgical outcome at one year post-operatively may be too early, as the participants in our study reported they were still improving. However, it may be important to identify those who are not recovering at the anticipated rate at an even earlier time in order to offer relevant resources, such as physiotherapy, nursing care, referral to the orthopaedic surgeon or psychologist, and to individualize care to improve the final outcome. A team of surgeons, physiotherapists and registered nurses should continuously provide the patient with information about common post-surgical recovery time and symptoms. This approach is in alignment with a recent review that suggested continuous information and education is essential for patients to be able to cope with pain and to understand their symptoms (See, Kowitlawakul, Tan, & Liaw, 2018).

For those participants who had reduced pain and improved function, their lives had returned to their baseline prior to the onset of OA. The feeling of loss that was described in the pre-operative interviews (Nyvang et al., 2016) was not a prominent theme in the present study. The silence of the knee could be interpreted as reuniting the body and self: from an object body—where the knee is a constant awareness—to a lived body—the silent knee—that was described by Gadow (Gadow, 1980). This concept of embodiment can also be applied for those who had remaining symptoms as they were struggling to accept to live with the awareness of the knee (Gadow, 1980).

Similar to our findings, Woolhead et al. (2005) found that most patients reported a good or excellent post-operative result despite some persistent pain (Woolhead et al., 2005). However, they proposed that reporting a good result may be more reflective of their desire to please the caregiver, keeping the elaborate answer about persistent pain to themselves (Woolhead et al., 2005). This might also be true for the participants in our study but our interpretation was rather that the participants could in fact be satisfied despite remaining symptoms: through surgery they had done what they could to relieve the symptoms. However, this highlights the complexity of the participants’ subjective experiences and the value of qualitative research, which has been stressed for patients with lower extremity trauma (Bernhoff, Bjorck, Larsson, & Jangland, 2016).

A central focus for the participants was the need to talk about pain, both pre- and post-operatively, as it is the main indication for performing the knee arthroplasty. Some participants reported not getting satisfactory post-operative pain relief and dissatisfaction with pain relief is associated with the risk of long-term post-surgical pain (Vadivelu et al., 2017). Besides the fact that persistent pain after surgery could be seen as a failure, pain is also a risk factor for a higher morbidity and mortality in cardiac and pulmonary complications post-operatively (Vadivelu et al., 2017).

Knee replacement surgery is a proven cost-effective procedure in those above 65 years of age (Daigle, Weinstein, Katz, & Losina, 2012). The median age in this study was 67 years and most participants had retired (9/12), as 65 years is the common retirement age in Sweden. We may assume that these participants, due to age, would retire regardless of the operation and that the timing of the surgical procedure was convenient in the patient perspective. We cannot draw any conclusion regarding the cost-effectiveness of surgery, but the participants improved with respect to pain and function, as measured by the KOOS (Figure 1) and these results are comparable to the scores reported in the Swedish knee arthroplasty registry (SKAR, 2016). Self-reported overall health, as measured with EQ-5D VAS, did not differ much before and after surgery (Table I). However, the post-operative scores are comparable to the scores in SKAR but not the pre-operative scores, which were higher in the present study (SKAR, 2016). These patients might have been healthier than the overall patient population, but we cannot draw any conclusions due to the small number of patients.

### Study limitations

The interviews and data analysis were conducted in Swedish and when translating quotes to English some information is at risk to be lost, which can be seen as a study limitation. However, the translated quotes were verified by a translator to ensure accuracy. Questions about major life events were not posed prior to the interviews and thus we have no knowledge if they occurred and further how they could affect the findings of this study, which is another possible study limitation.

### Conclusions and clinical implications

Some participants were not fully restored one year after surgery, despite being satisfied or not. Surgeons often inform patients that the recovery time after a knee arthroplasty is one year, which in light of this study, might be too short. Due to the nature of this qualitative study we cannot draw the conclusion that pain will change over time after the first year. However, we suggest that a follow-up after one year might identify those who need enhanced physical and psychological support to get the best possible outcome, whether it is to help patients accepting persistent symptoms or to continue striving towards a silent knee.
